# Highly contrasted responses of Mediterranean octocorals to climate change along a depth gradient

**DOI:** 10.1098/rsos.140493

**Published:** 2015-05-06

**Authors:** I. D. Pivotto, D. Nerini, M. Masmoudi, H. Kara, L. Chaoui, D. Aurelle

**Affiliations:** 1 Aix Marseille Université, CNRS, IRD, Avignon Université, IMBE UMR 7263, Marseille 13397, France; 2 Aix Marseille Université, CNRS, Université de Toulon, IRD, MIO UMR 7294, Marseille 13288, France; 3 Laboratoire Bioressources Marines – Université d'Annaba, Badji Mokhtar, BP 230, Oued Kouba, Annaba 23008, Algeria

**Keywords:** climate change, adaptation, acclimatization, *Eunicella cavolini*, Mediterranean sea, population genetics

## Abstract

Climate change has a strong impact on marine ecosystems, including temperate species. Analysing the diversity of thermotolerance levels within species along with their genetic structure enables a better understanding of their potential response to climate change. We performed this integrative study on the Mediterranean octocoral *Eunicella cavolini*, with samples from different depths and by means of a common garden experiment. This species does not host photosynthetic *Symbiodinium*, enabling us to focus on the cnidarian response. We compared the thermotolerance of individuals from 20 m and 40 m depths from the same site and with replicates from the same colony. On the basis of an innovative statistical analysis of necrosis kinetics and risk, we demonstrated the occurrence of a very different response between depths at this local scale, with lower thermotolerance of deep individuals. Strongly thermotolerant individuals were observed at 20 m with necrosis appearing at higher temperatures than observed *in situ*. On the basis of nine microsatellite loci, we showed that these marked thermotolerance differences occur within a single population. This suggests the importance of acclimatization processes in adaptation to these different depths. In addition, differences between replicates demonstrated the occurrence of a variability of response between fragments from the same colony with the possibility of an interaction with a tank effect. Our results provide a basis for studying adaptation and acclimatization in Mediterranean octocorals in a heterogeneous environment.

## 2. Introduction

Climate change constitutes an adaptive challenge for numerous species. This also provides an opportunity for a real-time investigation of biological adaptation from ecological and evolutionary points of view. The response to climate change will depend on the type and rate of adaptation to a fluctuating environment. The nature of the adaptive response, either through genetic adaptation (evolutionary response) or acclimatization (phenotypic buffering *sensu* Reusch [1]), is crucial for the future of species and biodiversity [1–5]. One might expect a potentially faster and less stable response for acclimatization than for genetic adaptation. The relative importance of these two responses can be investigated within a temporal framework through the study of well-characterized biological modifications linked with global warming [1,3]. An alternative approach relies on the study of differential responses in contrasted environments along clines or in extreme conditions reflecting the potential range of future conditions [5]. The study of marginal populations in extreme environments highlights the limits of the adaptive range of a species. The demonstration of local adaptation may provide some clues regarding the possibility of an evolutionary response to environmental change [1].

In heterogeneous landscapes, the evolution of genetic adaptation will depend on the relative strength of local selection and gene flow. High levels of gene flow compared to the scale of environmental heterogeneity can lead to migration load [6] or to the evolution of phenotypic plasticity [7]. Gene flow can also replenish the genetic diversity of marginal populations and facilitate the propagation of beneficial alleles [8,9]. Evaluating the respective levels of gene flow and of variations in adaptive abilities is therefore crucial to estimate the potential evolution of species facing climate change and is useful for management purposes [10]. Adaptive diversity and gene flow are not often studied simultaneously even for ecologically important species whose response will have community-level consequences. Such studies may be rendered more challenging by the different spatial scales at which contrasted responses to climate change can be observed within species. Very local environmental conditions can induce different responses but have only rarely been taken into account [11], even if such local effects might have important consequences at higher scales [12,13].

The effects of climate change are already clearly visible in the marine realm through acidification and temperature changes [14] and the potential adaptation of marine organisms to these changes should be studied in more detail [1,5]. This is especially the case for corals (here anthozoans) which are both ecologically important species and are impacted by bleaching or mortality events [15,16]. The comparison of coral populations from ecologically contrasted conditions, especially different thermal regimes, can inform us about their adaptive ability in the face of global warming. Mediterranean octocorals provide good ecological models in this context. In the last decades, they have been affected by mass mortality events linked with thermal anomalies with variable responses between species, populations and individuals within populations [17]. These species are also present at different depths corresponding to steep thermal gradients [18]. Experimental studies of the levels of thermotolerance in Mediterranean octocorals have provided contrasting results. In the non-symbiotic species *Corallium rubrum*, shallow populations (from more variable and stressful conditions) appeared to be more thermotolerant than deeper ones [18,19]. In the symbiotic gorgonian *Eunicella singularis*, shallow populations exhibited more stress signals than deeper ones [20,21] but with similar necrosis threshold temperatures [22]. This raises the question of the role of *Symbiodinium* in the adaptive response. The levels of genetic differentiation between depths were also different in these two cases, with a significant differentiation for *C. rubrum* [18,23,24] compared with no differentiation for *E. singularis* [22].

In order to better understand the evolution of adaptive differences under high gene flow conditions, and independently of the potential effect of *Symbiodinium*, we studied the thermotolerance of the non-symbiotic gorgonian *Eunicella cavolini*. *Eunicella cavolini* has been affected by mortality events [17] and preliminary results suggest that this species presents no genetic differentiation according to depth (M. Masmoudi, L. Chaoui, H. Kara, D. Aurelle 2014, unpublished data). If different responses are observed between depths, this might indicate either a plastic response or a genetic one but with potential migration load. In addition for these colonial organisms, the question of variability of response within colonies has not been studied, despite its potential importance in the colony's survival. We statistically analysed the rate and risk of necrosis and tested the null hypothesis of no thermotolerance differences between *E. cavolini* from two depths and between two replicate batches from the same colonies. Considering the genetic structure of octocoral species in this area, this provides a basis for discussion of the adaptive abilities of these species in a heterogeneous environment and in the face of seawater warming.

## 3. Material and methods

### 3.1 Model species

The yellow gorgonian *E. cavolini* (Koch, 1887) is an octocoral endemic from the Mediterranean Sea, but more common in the western than in the eastern Mediterranean [25]. It can be observed from less than 10 m to more than 100 m depth. This is a gonochoric species with internal fertilization and brooding, which can live more than 20 years [26]. Population genetics data indicate that this species mainly reproduces sexually, with rare cases of clonality (M. Masmoudi, L. Chaoui, H. Kara, D. Aurelle 2014, unpublished data).

### 3.2 Sampling sites

Individuals were sampled at two depths (20 m, range 18–22 m, and 40 m, range 38–41 m) along the same wall at Riou Island (Marseille, France; 43°10.370′ N, 5°23.420′ E). These two depths are characterized by different thermal regimes with higher and more variable summer temperatures at 20 m than 40 m. During 379 days of survey from June 2011 to July 2012, the maximum temperature reached 25.4°C at 20 m and 22.8°C at 40 m (F. Zuberer, Pythéas Institute 2014, personal communication; T-MedNet data, http://t-mednet.org/index.php). The minimum summer temperatures were similar between depths (13.9°C at 20 m and 13.7°C at 40 m in July 2012) and the mean temperature between June and September 2012 was 17.9°C at 20 m and 15.5°C at 40 m. From the end of autumn and during winter, the temperature is homogeneous between depths, with a minimum of 12.7°C in February 2012. For the common garden experiment, 20 individuals were sampled at each depth in March 2012, with a 13.2°C temperature at both depths. For the population genetic analyses, 30 additional individuals were sampled at each depth.

### 3.3 Common garden experiment

The 40 sampled colonies were placed in experimental aquariums at the Endoume Marine Station in Marseille. Each colony was divided into four fragments for this experiment: two replicates for the control condition and two for the stress condition. The replicates thus correspond to the same colony and to the same genotype. The mean length of the fragments was 2.24±0.13 cm (min. 1.2, max. 4). Stress and control conditions were separated in two tank systems and the replicates were placed randomly in three 24 l tanks for the control and four 18 l tanks for the stress conditions. The tanks corresponding to the same experimental condition (control or stress) were connected to the same water filtration system in order to focus on the variability linked to the different colonies or fragments. For each depth, replicates were separated in different tanks (two tanks for each batch of replicates) and replicates from different depths were mixed inside tanks. At the beginning of the experiment, the fragments were fixed on plastic supports and kept in open seawater at 16°C during 50 days for acclimation (electronic supplementary material, figure S1). This temperature is intermediate between the mean summer temperatures at each depth. After that step, the stress and control tanks were kept in a closed system with natural seawater and filtration, in order to accurately control the temperature conditions inside the tanks. The colonies were not fed during the whole experiment and air pumps provided oxygenation of the water. The control fragments were kept at 16°C during the following experiment. The thermal stress protocol was chosen after a preliminary experiment allowing determination of necrosis thresholds and based on the observed maximum temperature in summer (electronic supplementary material, figure S1). The final stress protocol comprised a two-stage temperature increase: from 16 to 23°C and from 23 to 27.5°C. The colonies were then kept at 27.5°C until the end of the experiment (electronic supplementary material, figure S2). The whole experiment lasted 90 days between 28 March and 25 June. Temperatures were recorded every 15 min with TidbiT data loggers (see the electronic supplementary material, figure S2) and each day necrosis levels were estimated as the percentage of necrosed tissue. Two types of necrosis were recorded: partial necrosis when living tissues presented a colour alteration or were partly detached from the colony; or full necrosis was recorded when the skeletal axis was visible (electronic supplementary material, figure S3). The percentages of partial and full necrosis for each fragment were estimated by eye with a ruler. The comparison of these measurements between three observers confirmed the repeatability of this approach. For the statistical analyses, we focused on the appearance of necrosis *sensu lato*: one fragment was considered affected if there was any sign of either partial or full necrosis.

### 3.4 Statistical analysis of necrosis levels and kinetics

In order to study the kinetics of necrosis, we focused on the proportion of fragments which did not present any necrosis (full or partial) in relation to time. We used this kinetics as a basis for understanding whether multiple causes of death or failure should be taken into account and how the probability of survival changed during the experimental time. The necrosis dynamics was processed through survival data analysis. Consider the survivor function *S*(*t*) which is defined as: *S*(*t*)=*Pr*(*T*>*t*), the probability that the necrosis time *T* (i.e. the time of first necrosis) is greater than some specified time *t*. The survivor function is related to the lifetime cumulated distribution function *F*_*T*_(*t*) with the relationship *F*_*T*_(*t*)=*Pr*(*T*≤*t*)=1−*S*(*t*).

This distribution function is connected to the density *f*_*T*_(*t*) of *T* as, by definition $F_{T}(t)=\int_{-\infty }^{t}f_{T}(\tau )\text{d}\tau$. Thus, *S*(*t*) is a decreasing function of time, starting from 1 (all individuals are not affected) and going to 0 (all individuals are necrosed) as *t* goes to infinity. The shape of *S*(*t*) is strongly dependent on the shape of a density *f*_*T*_(*t*). Without assumption upon the form of *f*_*T*_(*t*), we fitted the observed data with a Weibull distribution:

$$F_{T}(t)=1-{\text{e}}^{-(t/\theta {)}^{\lambda }}.$$

This distribution is widely used in reliability and life data analysis because of its versatility. The shape of the Weibull distribution is controlled by two positive parameters, the shape λ and the scale *θ*, and can be used to model various life behaviours [27]. A shape higher than 1 indicates that necrosis accelerates with time and higher scale values indicate a faster development of necrosis. Estimates of (*θ*,λ) have been obtained by nonlinear regression (Nelder–Mead simplex method [28]) by minimizing the usual Euclidean distance between the Weibull distribution and the cumulated proportion of necrosed fragments for each sample (two replicates at 20 m, two replicates at 40 m). On the basis of these parameter estimates, the survivor function was displayed given a probability of survival beyond time *t* as well as associated 95% confidence intervals for the regression and binomial likelihood intervals for the data. Under normality assumptions, it is possible to draw confidence ellipses in the parameter space and then to compare samples using classical *p*-values. As an illustrative example of other possible fits, monotonic constrained smoothing splines have also been superimposed on the initial data (electronic supplementary material, figure S4; [29]).

We then considered the cumulative hazard function, which in the case of a Weibull distribution is given by *H*_*T*_(*t*)=(1/*θ*^λ^)*t*^λ^. This function is a measure of risk and increases with time: the greater the value of *H*_*T*_(*t*), the greater the risk of necrosis by time *t*. This allowed comparison of the different groups (depths and replicates).

### 3.5 Population genetics analyses

The 20 individuals used in the experiment and the 30 additional individuals from each depth were genotyped with nine microsatellite loci (electronic supplementary material, table S1). Genclone v. 2.0 [30] was used to search for identical multilocus genotypes (MLG) and to compute the probability *p*-sex of obtaining identical MLGs through sexual reproduction. We observed one and four repeated MLGs at 20 and 40 m, respectively, with *p*-sex≤0.0001. They corresponded to one MLG present both in the experiment and in the additional genetic sampling: this most probably corresponded to the repeated sampling of the same colonies and we discarded the corresponding individuals from the additional genetic sampling. Linkage disequilibrium between loci was tested with the log-likelihood ratio statistic implemented in Genepop v. 4.2 [31]. Allelic richness and observed and expected heterozygosities were computed with Genepop as 1-Qintra and 1-Qinter, respectively. The estimators of *F* statistics *F*_IS_ and *F*_ST_ [32] were computed with Genepop. We tested the null hypothesis of panmixia with a global Hardy–Weinberg test implemented in Genepop by considering the alternative hypothesis of heterozygote deficit. The genetic differentiation between samples was tested by an exact test procedure [33] implemented in Genepop with default parameters. An analysis of molecular variance (AMOVA, [34]) was performed to compare the two depths, 20 and 40 m, and the experimental and additional samples for each depth. The AMOVA was computed on the basis of *F*_ST_ and *R*_ST_ with Arlequin v. 3.5.1.3 [35]. Two fixation indices were considered: *F*_SC_ which compares samples within groups (here within depths) and *F*_CT_ which compares groups of samples (here different depths). The AMOVA was repeated by excluding Ever009. As a complementary analysis of genetic structure, a Bayesian clustering analysis has been performed (see details in the electronic supplementary material).

## 4. Results

### 4.1 Comparative thermotolerance

**Figure 1.**
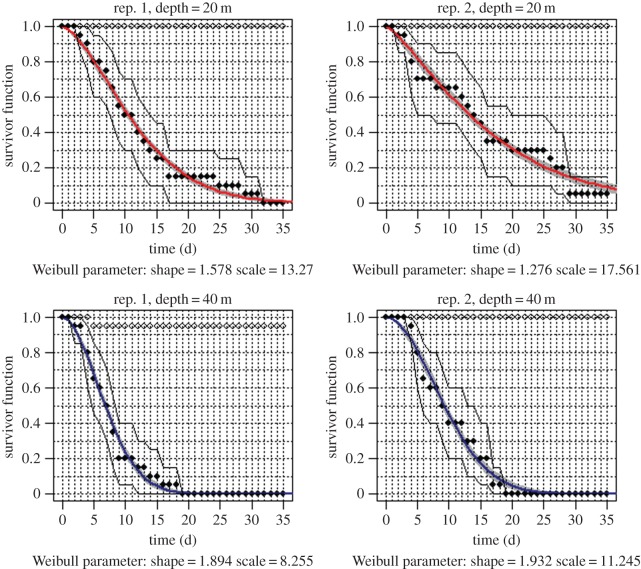
Survivor (proportion of individuals without necrosis) as a function of time. Time 0 corresponds to the beginning of experimental stress (23°C, see the electronic supplementary material, figure S2). Rep: replicate number for each depth. Black and white dots: observed data for the stress and control conditions, respectively. Red line: Weibull survivor function with the corresponding parameters indicated below each graph and the 95% confidence interval in grey. Black lines: binomial likelihood intervals for the data. For the sake of clarity, the different samples are presented on separate panels.

During the whole experiment, almost no sign of necrosis was observed in the control condition at 16°C in specimens from either depth. Only one 40 m fragment appeared necrosed at its base, with 13% of necrosis from the 5th day of the experiment, and this level did not change until the end of the experiment (figure 1). In the stress condition, the first necrosis was observed after 2 days at 27.5°C (electronic supplementary material, figure S4). Necrosis appeared sooner for 40 m individuals compared with 20 m individuals (mean time of first necrosis: 9 days for 40 m and 14 days for 20 m). Starting from the 19th day of the experiment, all 40 m fragments were necrosed. At the end of the experiment, after 36 days at 27.5°C, 2.5% of 20 m fragments were still intact. The necrosis curves also suggested some differences between replicates from the same depth.

The fit of a Weibull distribution allowed a good description of the observed data (figure 1). This indicated clear differences between depths with a Weibull shape of 1.578 and 1.276 for the two replicates at 20 m and 1.894 and 1.932 at 40 m. This indicates that necrosis accelerates with time. The scale varied between 13.27 and 17.561 at 20 m and 8.255 and 11.245 at 40 m, indicating a faster effect of necrosis per time unit in the case of individuals from 40 m than individuals from 20 m.

**Figure 2.**
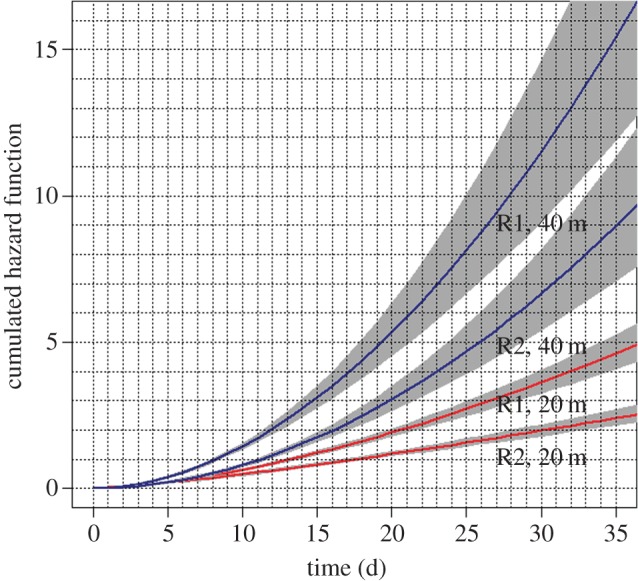
Patterns of change in necrosis risk through time. The curves represent the cumulative hazard function (see text for details).

**Figure 3.**
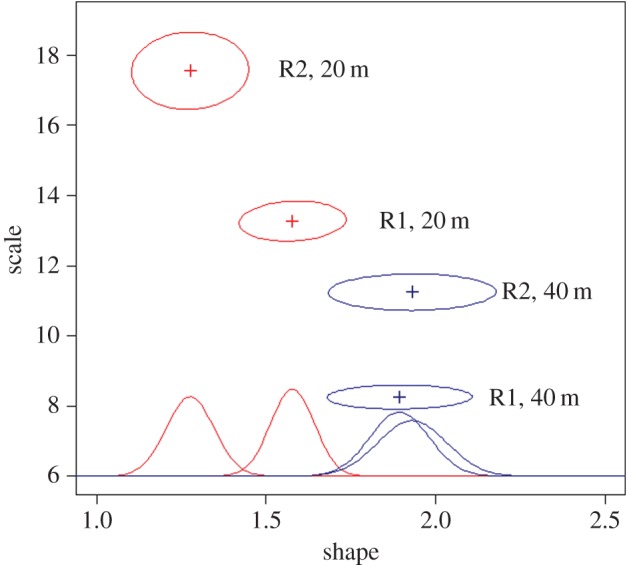
Ninety-five per cent confidence ellipses from the nonlinear fit of Weibull distribution under normality assumptions. The shape of the ellipses reflects an independence between shape and scale parameters (weak covariances). The display of the marginal distributions for the shape parameter highlights differences between the 20 m and 40 m samples by computing *p*-values between replicates.

Cumulative hazard functions indicated that the risk of necrosis increased with time (figure 2). A clear difference in necrosis levels was also evident between colonies collected from 20 m and 40 m. The risk was higher and increased much faster at 40 m than at 20 m. The risk also appeared different between replicates but was always lower at 20 m than 40 m. A display of the 95% confidence ellipses of the shape and scale parameters showed no overlap between depths nor between replicates, thus demonstrating significant differences between depths and replicates (figure 3). The replicates 1 at 20 m and 2 at 40 m were close together, though separated, in the parameter space. The shape was lower at 20 m than 40 m (*p*=0.022 for the closest comparison between 20 m and 40 m; figure 3, marginal distributions), with overlapping confidence intervals for the two 40 m replicates but significant differences between the two replicates at 20 m (*p*=0.013). An analysis of survivor curves per tank and per depth indicated that fragments from 20 m in the C5 tank showed necrosis sooner and faster than fragments from the same depth in other tanks (electronic supplementary material, figure S5). This was not observed for the fragments from 40 m in this C5 tank when compared with other fragments from the same depth.

### 4.2 Population genetics

Similar levels of genetic diversity were observed for the 20 m and 40 m samples (including experimental and *in situ* samples) with *H*_exp_=0.56 at 20 m and 0.55 at 40 m (electronic supplementary material, table S2). The number of alleles per locus and per depth ranged from 2 to 8, with a mean value over the nine loci of 5.2 at 20 m and 5.4 at 40 m (electronic supplementary material, table S2). Tests of adequation to Hardy–Weinberg proportion were not significant in most cases, apart from locus EC24 at both depths and S14 at 20 m for which deficits of heterozygotes were observed. Significant heterozygote deficits were also observed for the multilocus test at each depth. Four alleles were private to one depth: two at 20 m and two at 40 m with a maximum frequency of these private alleles reaching 0.04 (data not shown). The test of linkage disequilibrium between loci revealed one significant association for both depths, between loci C21 and EC32 (*p*=0.014 and <0.001 at 20 m and 40 m, respectively; data not shown).

The differentiation test between experimental and additional *in situ* samples was not significant at 20 m (*F*_ST_=0.007; *p*=0.669; table 1) and slightly significant at 40 m (*F*_ST_=0.013; *p*=0.031). The differentiation between depths, by grouping experimental and *in situ* samples for each depth, was not significant (*F*_ST_=0.001; *p*=0.24). Single locus genic differentiation tests between depths were non-significant apart from locus Ever009, for which a significant differentiation was observed (*F*_ST_=0.02; *p*=0.021). The AMOVA indicated a significant differentiation between samples inside each depth with *F*_ST_ like analysis (*F*_SC_=0.011; *p*=0.043) but not with *R*_ST_ (*F*_SC_=−0.027; *p*=0.992; electronic supplementary material, table S3). The differentiation between depths was not significant with the AMOVA (*F*_*CT*_=−0.004 and 0.016 based on *F*_ST_ and *R*_ST_, respectively). If locus Ever009 was omitted from AMOVA, no differentiation was significant, neither between depths nor between samples inside depths. The Bayesian clustering analysis did not reveal any genetic structure, which agrees with the lack of differentiation (electronic supplementary material, figure S6).

**Table 1.** *F*_ST_ values between depths per locus. (Italicized value corresponds to significant differentiation between the two depth samples based on an exact test (*p*=0.021 for Ever009).)

locus	C21	C30	C40	S14	Ever007	Ever009	EC17	EC24	EC32	all
*F*_ST_	−0.006	−0.008	0.001	−0.006	0.007	*0.02*	−0.004	0.016	−0.006	0.001

## 5. Discussion

### 5.1 Necrosis thresholds and kinetics of *Eunicella cavolini*

Our results underline the thermotolerance abilities of the yellow gorgonian in the northwestern Mediterranean Sea. For the 20 m individuals, after 28 days of exposure at 27.5°C, 15–20% of the fragments (depending on the replicate) displayed less than 5% necrosis levels. This demonstrates the existence of highly thermotolerant individuals despite the fact that this temperature is rarely or never encountered by the populations tested here. This could suggest that shallow populations may live far from their thermotolerance limits but such experimental results can differ from *in situ* thermotolerance levels. Under natural conditions in 2003, mortality events were observed for three octocoral species, including *E. cavolini*, with maximum summer temperatures around 22°C at 20 m [17]. Such discrepancy between ecological surveys and experimental results has already been observed for other Mediterranean octocorals [19,20,36]. This may be linked to the length of the exposure: *in situ* mortality events often took place at the end of summer, after the repetition of several thermal stress episodes of different intensity and duration. The date of the experiment may be important as well. Gene expression studies revealed different responses to thermal stress depending on the month for the Mediterranean red coral *C. rubrum*, with a potentially more efficient response in March compared with June [18]. Our experiment started at the end of winter, whereas summer is a more stressful season because of low food availability [37]. It should be noted here that even without feeding the colonies, we observed high levels of thermotolerance. The occurrence of thermodependent pathogens could also induce necrosis at lower temperature, as demonstrated for the Mediterranean octocoral *Paramuricea clavata* [38]. For the congeneric *Eunicella verrucosa*, diseased colonies also show modifications of bacterial communities [39]. The necrosis kinetics evidenced here with the Weibull law is compatible with deteriorating physiological conditions at sublethal temperatures or with the development of pathogens following a decline of defences [17]. Here, the necrosis risk also increased strongly with the duration of exposure: this is consistent with *in situ* observations of mortality events following episodes of stable warm temperatures. We demonstrated that this risk is higher and increases more quickly for 40 m than 20 m individuals.

### 5.2 Contrasted responses to thermal stress among species of the genus *Eunicella*

The comparison of the experimental thermotolerance results obtained in this article and in previous studies provides a basis for discussion of the differences in adaptation to local thermal regime and depth for different *Eunicella* species. First, at the interspecific level, the necrosis threshold observed here for *E. cavolini* is similar to that observed for *E. singularis*, in the same area and depth range [22]. Nevertheless, the intraspecific pattern of thermotolerance levels is different between these two species. Whereas we clearly demonstrated here higher theromotolerance for shallow populations of *E. cavolini* compared with deep ones, in *E. singularis* deep populations appeared more thermotolerant than shallow ones for a similar depth range [20,21]. Nevertheless, there were no differences in necrosis threshold between depths for *E. singularis* [22]. The differences between these species may be linked to the different experimental protocols used: for example, in these experiments, *E. singularis* colonies were fed [20,22] which was not the case here. A dedicated experiment would be required to test whether the interaction between food and thermal stress can induce such different patterns of thermotolerance. The duration of thermal stress was also different between these studies but this parameter is less likely to reverse the patterns of thermotolerance between depths. Finally, the presence of *Symbiodinium* symbionts in *E. singularis*, in contrast to *E. cavolini*, is probably a major factor explaining these different results. As suggested by Ferrier-Pagès *et al.* [20], shallow populations might be more exposed to free radicals because of higher photosynthetic activities. This could exacerbate the impact of thermal stress at shallow depths, and this would be only partially compensated by the host acclimatization in *E. singularis*. Photosynthetic symbionts might be advantageous as a way of overcoming the low food summer season [40] but they could sometimes have detrimental effects. The interaction with *Symbiodinium* may not always be mutualistic [41]. The close relatives of *E. singularis*, *E. cavolini* and *E. verrucosa* [42], do not harbour *Symbiodinium*: therefore, this symbiosis may be relatively recent, which raises the question of its stability and its adaptive consequences.

### 5.3 Contrasted responses to thermal stress: intraspecific level and interaction between adaptation and gene flow

Our experimental results uncovered highly contrasting thermotolerance response between depths in *E. cavolini*. Such differential phenotypic buffering is in sharp contrast with the low and non-significant genetic differentiation observed between depths (*F*_ST_=0.001). A low genetic structure was also observed for *E. singularis*, with five microsatellite loci (non-significant differentiation; no information about *F*_ST_ [22]). Two other octocorals, *C. rubrum* and *P. clavata*, displayed significant differentiation along the same depth gradient (*F*_ST_=0.06 and 0.03, respectively; [23,43]). *Corallium rubrum* also displayed a contrasted response to thermal stress between depths, and thus for different genetic pools [18,19,44]. Our study is then, to our knowledge, the first demonstration of such clear thermotolerance differences between depths within the same genetic pool and without potential interference effect from *Symbiodinium*.

Two hypotheses should be considered to explain the observed differential response to thermal stress in this context. The genetic hypothesis states that these differences are genetically based and locally selected (i.e. genetic adaptation). According to the acclimatization hypothesis, such differences could be environmentally induced during individual life history and maintained for example through epigenetic processes. Concerning *E. cavolini*, the low observed differentiation implies regular gene flow between depths. In the case of the genetic hypothesis, gene flow would induce a migration load at each generation and this could limit the action of local selection [6]. Precise estimates of gene flow are required to evaluate the interaction between migration and local selection. Moreover, the reduced number of loci analysed here do not allow us to reject the possibility of genetic adaptation determined by other genomic regions. Theoretical predictions indicate the possibility of the persistance of locally adaptive polymorphisms despite gene flow, for example with fewer loci with larger effect [45]. For *E. cavolini*, the adaptation to the different thermal regimes could also correspond to acclimatization or to a combination of the genetic and non-genetic effects as for the tropical coral *Acropora hyacinthus* [13]. Other Mediterranean octocorals present different thermotolerance levels but in conditions of higher genetic structure, which could be more favourable to local adaptation. This is the case for *C. rubrum* along the same depths as the ones studied here. At higher distances, contrasted responses have been demonstrated between Catalonian and Balearic individuals for *E. singularis* [36]: here, the geographical and genetic distance between populations (distinct genetic clusters; [46]) make genetic adaptation more probable.

Additional studies, both experimental and genetics, are required to test these hypotheses. Our observation of a wide range in necrosis time (figure 1) indicates a diversity of response between individuals from the same depth. This suggests the possibility of genetic differences in acclimatization ability. Inter-generational common garden experiments would be the best way to test this, but this is very difficult for these long-lived species. Indirect evidence might come from the study of genetic×environment associations based on a high number of genetic markers (e.g. [47]). Transcriptomic studies can also be used to search for stable adaptive patterns which might be genetically determined [13,48,49].

The observed differences between replicates suggest either a tank effect or thermotolerance differences within colonies. The marginal distributions of the shape parameter of the Weibull hazard function showed no difference between replicates at 40 m but significant differences between replicates at 20 m (figure 3). The shape parameter is the most important in the Weibull hazard function as it mainly controls the rate of increasing risk. The replicates were randomly distributed between tanks connected to a common water system but different replicates were present in different tanks and the survivor curves indicated a difference for 20 m fragments in one tank. Even if one could expect an exposure to the same water parameters, including temperature, between tanks, a tank effect cannot be excluded. Nevertheless, this tank effect would have to be specific of the 20 m depth as it was not observed at 40 m. Another interpretation would be that these differences are due to an intra-colony variability of response. This has been demonstrated for several hexacorals, for example depending on location within the colony [50,51]. Here, we did not have any information on the original orientation of the fragment but such differences could be linked to differences in food availability, in the age of the branches or to previous injuries from predators or pathogens, even if colonies were healthy when sampled. This intra-individual variability would provide an additional level of adaptation by allowing the survival of a colony through differential impact of thermal stress along its branches. Additional tests taking into account the orientation of the fragments *in situ* and their position in the colony will be required to go further with this line of inquiry. They should be performed in common tanks. Our results indeed underline the interest of replicates from the same individuals for experimental studies on colonial organisms.

### 5.4 Thermotolerance and response to environmental changes

As a consequence of global warming, Mediterranean octocorals will face more stressful thermal regimes. For these species, a northward range shift is difficult because of the geography of the Mediterranean Sea. Their persistence will rely either on deeper populations acting as refugia (the deep refugia hypothesis; [52]) or on potential change in the response to thermal stress. With regards to the deep refugia hypothesis, we evidenced high gene flow between depths which could allow recolonization from deep populations less impacted by climate change. Our results underline the adaptive abilities of *E. cavolini* with potentially both acclimatization ability and adaptive genetic diversity, as in the tropical coral *A. hyacinthus* [13]. For *E. cavolini*, acclimatization should partly buffer the expected climate changes. Acclimatization through time has also been suggested for tropical corals but its long-term stability remains to be studied [53]. Non-genetic inheritance or genetic assimilation could contribute to the persistence of such acclimatization through successive generations [54] but temporal surveys and dedicated experiments are required to test these hypotheses. Acclimatization can also be limited by the interactions with pathogens or by physiological trade-offs [18]. With regards to temporal genetic adaptation, it will depend on the rate and strength of climate change compared to generation time. Octocorals can present sexual maturity after several years (e.g. [55]). Generation time will then constitute a limiting factor to an evolutionary response to climate change for these species. The evolutionary response also depends on the proportion of genetic variance in response diversity which remains to be estimated. In *E. cavolini*, connectivity levels between contrasted environments would be favourable to the spread of alleles allowing genetic adaptation [10]. Nevertheless, the rate of the current change and other human pressures such as acidification and habitat destruction could hinder the possibilities of adaptation and restrict them to a subset of anthozoan species [56].

## 6. Conclusion

The diversity of response to thermal stress in temperate octocorals underlines the need to take into account local diversity to study the potential response to climate change. Acclimatization and genetic diversity can induce very different thermotolerance levels along small vertical distances. The modular organization of these organisms provides an additional level of adaptation, with differences between parts of the same colony. These results open the way for transcriptomic and genomic studies of adaptation in temperate octocorals which are impacted by climate change. Non-symbiotic species are good alternative models to study adaptive processes in corals by focusing on the host (cnidarian) response. It is also important to take into account a dynamic view of the thermotolerance of species, populations and individuals for a better understanding of the potential fate of coral communities in the face of climate change. From a management point of view, our results demonstrate the need to protect shallow octocoral populations which are both the most thermotolerant, but also exposed to the harshest thermal conditions and human pressures.

## Supplementary Material

Microsatellite genotyping : locus characteristics and PCR conditions Table S1: amplification conditions and origin of the nine microsatellite loci Table S2: microsatellite diversity by depth Table S3 : results of the Analysis of Molecular Variance with nine microsatellite loci. Figure S1: protocol of thermal stress (A) and surveys of necrosis (B) for the preliminary experiment in stress condition Figure S2 : experimental protocol for the main thermal stress experiment Figure S3 : examples of necrosis on Eunicella cavolini during the preliminary experiment Figure S4: experimental data and constrained regression splines for the different proportions of necrosed individuals through time

## Data accessibility

Population genetics data and detailed necrosis data (partial and full necrosis for all samples and experimental times) are available in Dryad: (doi:10.5061/dryad.q45n7).
