# Supplementary material for: Highly contrasted responses of Mediterranean octocorals to climate change along a depth gradient
Source: R Soc Open Sci. 2015 May 6;2(5):140493. doi: 10.1098/rsos.140493 (PMC4453260; doi:10.1098/rsos.140493)

**Supplementary material**

**Microsatellite genotyping : locus characteristics and PCR conditions**

Total DNA was extracted either with QIAamp® DNA Mini Kit or with Macherey Nagel NucleoSpin ® kits on epMotion ® 5075 VAC automated pipetting system from Eppendorf. Depending on the loci (see Table S1), PCR were performed either separately of by multiplex PCR. The single locus PCR were performed with 1 µL of DNA template, 1X reaction buffer, 0.5 mM dNTP, 2.5 mM MgCl_2_, 1 µM of each primer, 0.05 U of GoTaq® Flexi DNA Polymerase (Promega) for a final volume of 10 µL. The PCR program was: 3 min at 94°C followed by 30 cycles with 1 min at 94°C, 1 min at annealing temperature and 1 min at 72°C; a final extension of 5 min at 72°C was added. The multiplex PCR were performed in 10 µL final volume with primers at 2µM with the Qiagen Type-it microsatellite PCR kit. The PCR program was: 5 min at 95°C followed by 30 cycles with 30 s at 95°C, 1 min 30 s at annealing temperature and 30 s at 72°C. PCR products were analyzed on an ABI 3130 genetic analyzer (Life Technologies) and the genotypes were determined with the GeneMapper v.3.5 software (Life Technologies).

**Table S1: amplification conditions and origin of the nine microsatellite loci**

| Locus | Annealing temperature | Mutliplex / simplex | Reference |
| --- | --- | --- | --- |
| C21 | 55°C | Simplex | 1 |
| C30 | 62°C | Simplex | 1 |
| C40 | 60°C | Simplex | 1 |
| S14 | 64°C | Simplex | 1 |
| Ever007 | 57°C | Multiplex 1 | 2 |
| Ever009 | 57°C | Multiplex 1 | 2 |
| EC17 | 57°C | Multiplex 2 | 3 |
| EC24 | 57°C | Multiplex 2 | 3 |
| EC32 | 57°C | Multiplex 2 | 3 |

1.Abdoullaye D *et al*. 2010 Permanent genetic resources added to molecular ecology resources database 1 August 2009–30 September 2009. *Mol. Ecol. Resour.* **10**, 232–236. (doi :10.1111/j.1755-0998.2009.02796.x)

2. Holland L, Dawson D, Horsburgh G, Krupa A, Stevens J. 2013 Isolation and characterization of fourteen microsatellite loci from the endangered octocoral *Eunicella verrucosa* (Pallas 1766). *Conser. Genet. Resour*, **5**, 825–829. (doi :10.1007/s12686-013-9919-3)

3. Masmoudi M, Aurelle D, Hammami P, Topçu NE, Kara H, Chaoui L (in prep.)

**Table S2: microsatellite diversity by depth. The following parameters are indicated for each locus and avor all loci: allelic richness, observed heterozygosity (Hobs), expected heterozygosity (Hexp), F_IS_. Asterisks indicate significant deviation from panmixia: * p < 0.5; ** p < 0,01; *** p < 0.001. Sample sizes after removal of repeated MLGs: 51 for 20 m and 47 for 40 m.**

| Locus | Depth | 20 m | 40 m |
| --- | --- | --- | --- |
| C30 | Allelic richness | 2 | 2 |
|  | Hobs | 0.18 | 0.21 |
|  | Hexp | 0.17 | 0.19 |
|  | F_IS_ | -0.09 | -0.11 |
| C40 | Allelic richness | 4 | 5 |
|  | Hobs | 0.59 | 0.72 |
|  | Hexp | 0.63 | 0.69 |
|  | F_IS_ | 0.06 | -0.05 |
| C21 | Allelic richness | 6 | 6 |
|  | Hobs | 0.69 | 0.68 |
|  | Hexp | 0.64 | 0.69 |
|  | F_IS_ | -0.08 | 0.01 |
| S14 | Allelic richness | 9 | 9 |
|  | Hobs | 0.64 | 0.71 |
|  | Hexp | 0.85 | 0.84 |
|  | F_IS_ | 0.24*** | 0.16 |
| Ever007 | Allelic richness | 4 | 4 |
|  | Hobs | 0.20 | 0.09 |
|  | Hexp | 0.19 | 0.08 |
|  | F_IS_ | -0.06 | -0.02 |
| Ever009 | Allelic richness | 6 | 7 |
|  | Hobs | 0.78 | 0.81 |
|  | Hexp | 0.81 | 0.75 |
|  | F_IS_ | 0.03 | -0.09 |
| EC17 | Allelic richness | 5 | 5 |
|  | Hobs | 0.63 | 0.53 |
|  | Hexp | 0.55 | 0.56 |
|  | F_IS_ | -0.14 | 0.06 |
| EC24 | Allelic richness | 3 | 4 |
|  | Hobs | 0.31 | 0.24 |
|  | Hexp | 0.43 | 0.33 |
|  | F_IS_ | 0.28*** | 0.29** |
| EC32 | Allelic richness | 8 | 7 |
|  | Hobs | 0.78 | 0.78 |
|  | Hexp | 0.79 | 0.81 |
|  | F_IS_ | 0.01 | 0.04 |
| Mean over loci | Allelic richness | 5.2 | 5.4 |
|  | Hobs | 0.53 | 0.54 |
|  | Hexp | 0.56 | 0.55 |
|  | F_IS_ | 0.05*** | 0.03* |

**Table S3 : results of the Analysis of Molecular Variance (AMOVA) with nine microsatellite loci (a) or eight loci (b, by omitting locus Ever009). The results are based on F_ST_ and R_ST_ like analyses and the significance of the different statistics has been tested with 1 000 permutations, significant values are indicated in bold. The analysis compared the two experimental samples (n = 20 at each depth) and the two additional in situ samples (n = 31 at 20 m and n = 27 at 40 m). For the AMOVA, the experimental and in situ samples were grouped by depth. F_ST_ indicates overall population differentiation, F_SC_ indicates differentiation between samples inside depths and F_CT_ indicates differentiation between depths.**

1. All loci

|  | F_ST_ | F_SC_ | F_CT_ |
| --- | --- | --- | --- |
| F_ST_ like | **0.007** | **0.011** | -0.004 |
| P value | **0.036** | **0.043** | 1 |
| R_ST_ like | -0.010 | -0.027 | 0.016 |
| P value | 0.875 | 0.992 | 0.338 |

1. Without Ever 009

|  | F_ST_ | F_SC_ | F_CT_ |
| --- | --- | --- | --- |
| F_ST_ like | 0.001 | 0.008 | -0.006 |
| P value | 0.221 | 0.102 | 1 |
| R_ST_ like | -0.011 | -0.024 | 0.013 |
| P value | 0.908 | 0.981 | 0.369 |

**Preliminary thermal stress experiment : the aim of this experiment was to evaluate which temperature should be used for the main experiment.**

The aim of the preliminary experiment was to define the adequate temperature allowing to study different thermotolerance levels in *Eunicella cavolini*: we needed to define a protocol of thermal stress which would induce a visible response (necrosis) with potential differences between colonies from different depths. For this preliminary experiment, six colonies have been sampled on Riou Island (Marseille) at 20 m and 40 m depth, in January, with a seawater temperature around 14°C. Each colony has been fragmented in four fragments : two for the control condition and two for the stress condition. After an acclimatization at 17°C in two steps, the control condition was maintained at 17°C and the stress condition corresponded to a gradual increase up to 28°C (Fig. S1A).

Necrosis appeared after 25 and 23 days of stress, or 10 and 8 days at 28°C, for colonies from 20 m and 40 m, respectively. After 36 days of experiment, 7 and 8 fragments over 12 presented necrosis for colonies from 20 m and 40 m, respectively (Fig. S1B). We therefore choosed 28°C as a final temperature for the main experiment.

**Figure S1: protocol of thermal stress (A) and surveys of necrosis (B) for the preliminary experiment in stress condition**


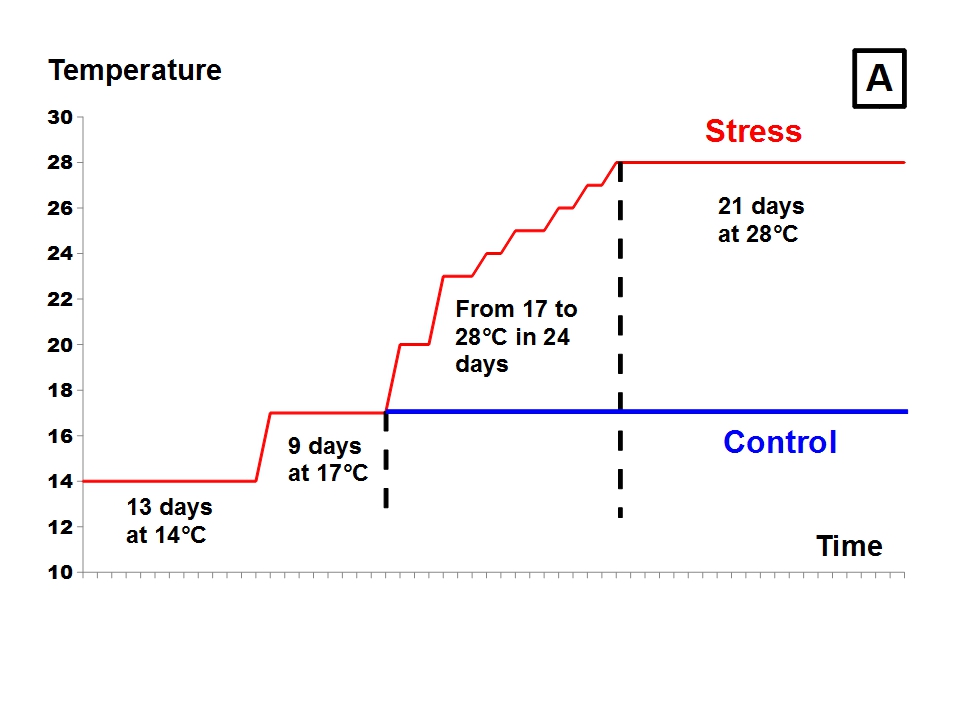


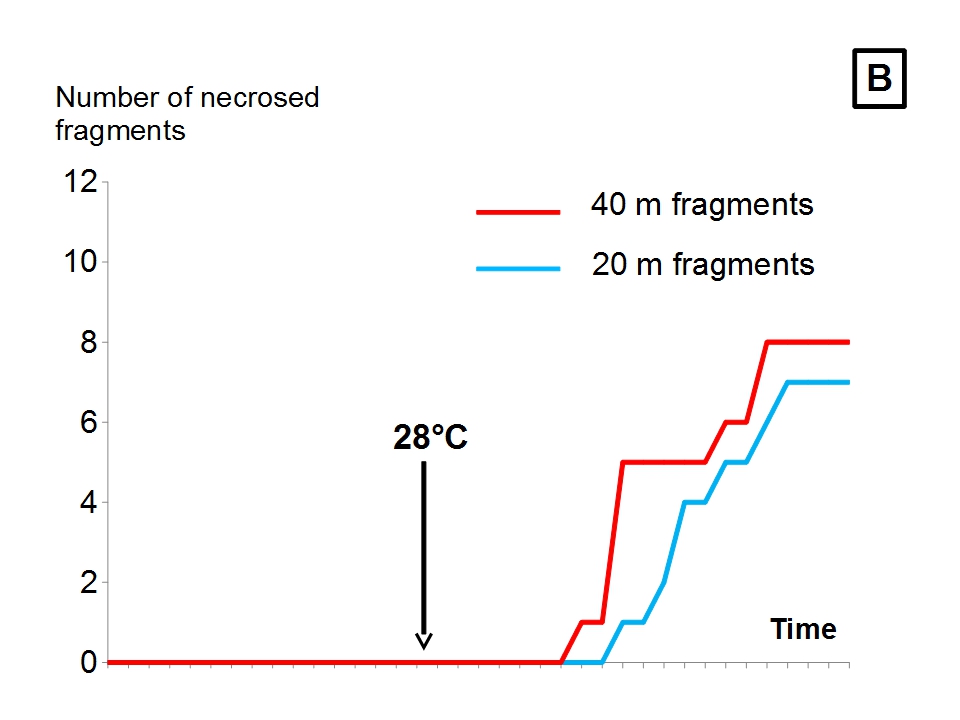


**Figure S2 : experimental protocol (a) for the main thermal stress experiment and observed temperatures (b): the real temperature data are based on Tidbit data loggers with 15 min measures interval**

1. **Experimental protocol:**

**
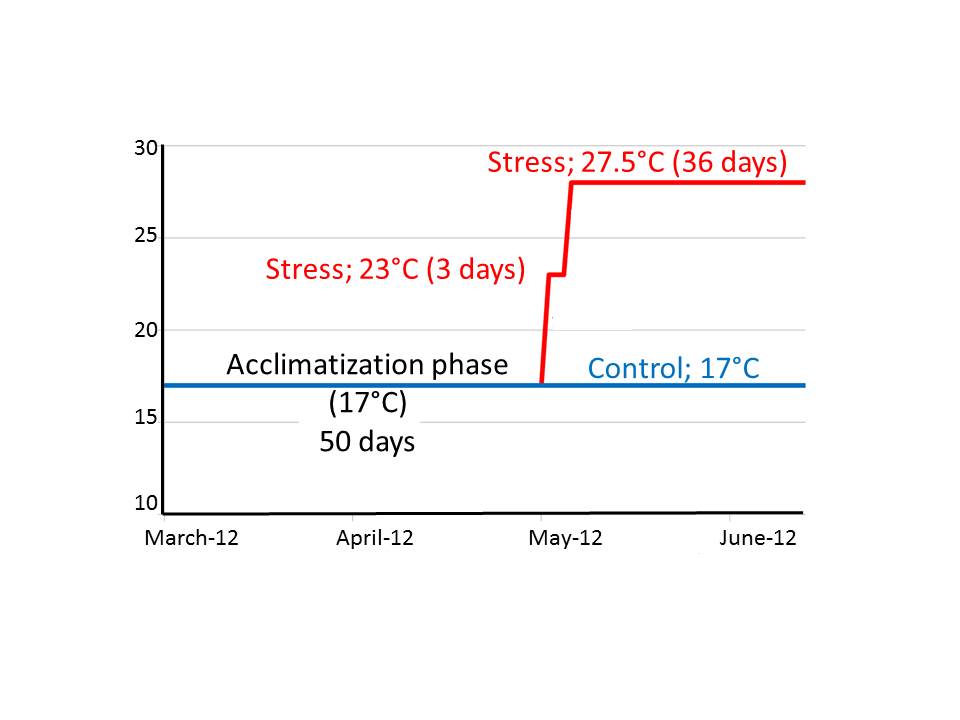
**

1. **Observed temperatures in the aquariums; red: stress condition, blue: control condition**

**Figure S3 : examples of necrosis on *Eunicella cavolini* during the preliminary experiment**


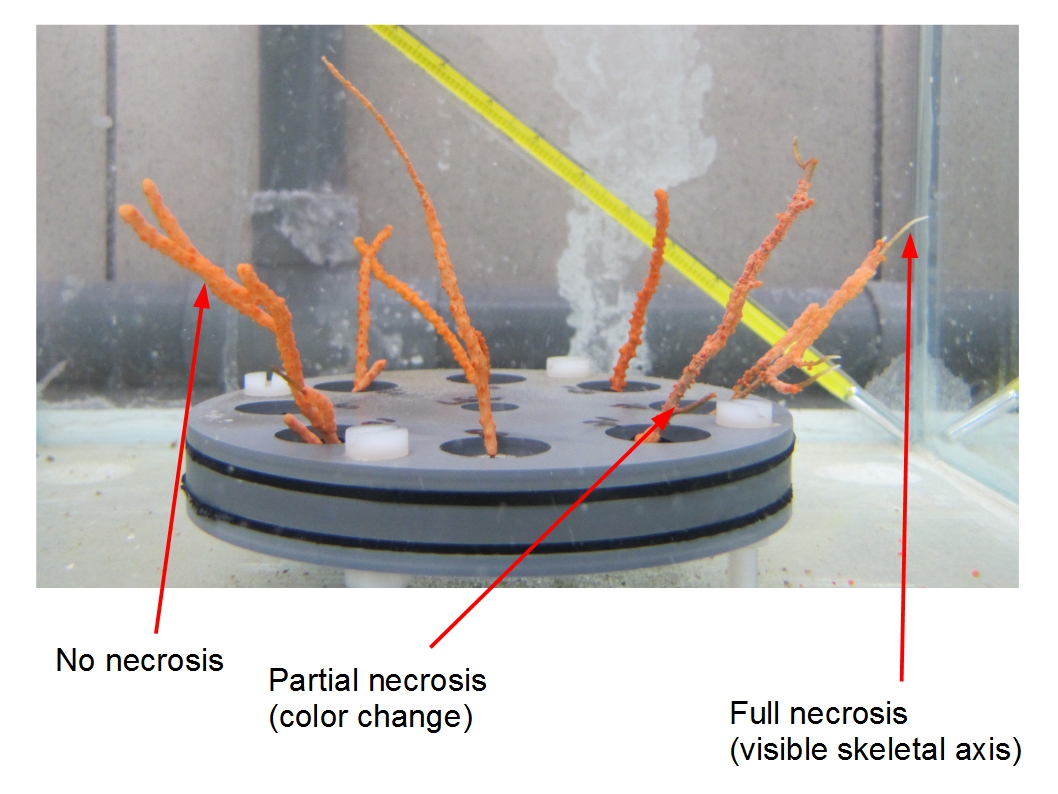


**Figure S4: experimental data and constrained regression splines for the different proportions of necrosed individuals through time.**

These strictly increasing functions fitted on the experimental data show the response of the different sample groups through time. Clear differences between depths appeared around the 19^th^ day. In order to better appreciate these differences a parametric Weibull model has been fitted (see main text). The samples codes indicate the replicate number followed by the depth of origin.


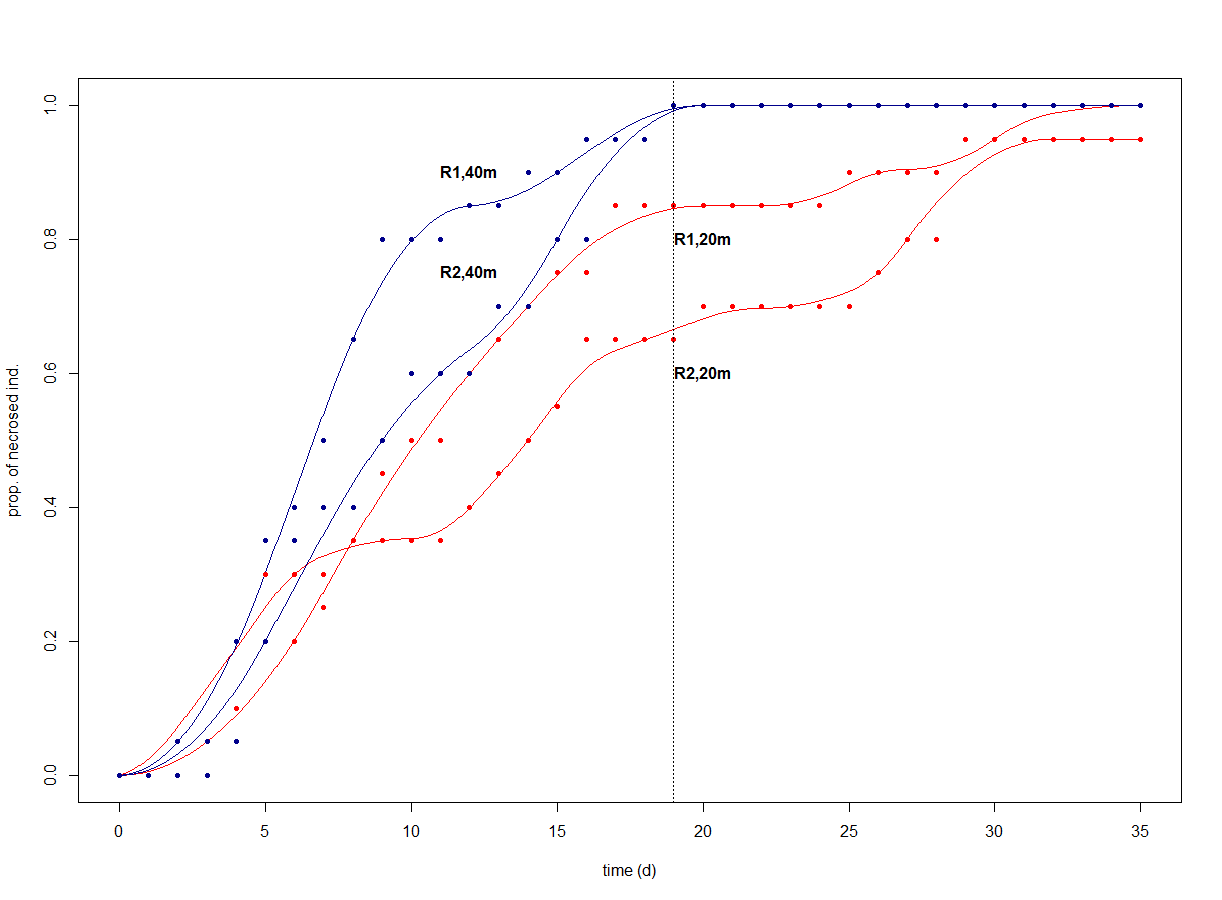


**Figure S5: survivor (proportion of individuals without necrosis) as a function of time per replicates and tanks**. Time 0 corresponds to the beginning of experimental stress (23°C, see Fig. S2). Rep: replicate number for each depth. For each depth, the first batch of replicates was placed in the C2 and C5 tanks and the other batch was in the C1 and C3 tanks. Black dots and white dots: observed data for the stress and control conditions respectively. Black line inside the grey area: Weibull survivor function with the 95% confidence interval in grey. Black lines: binomial likelihood intervals for the data. For the sake of clarity the different samples are presented on separate panels


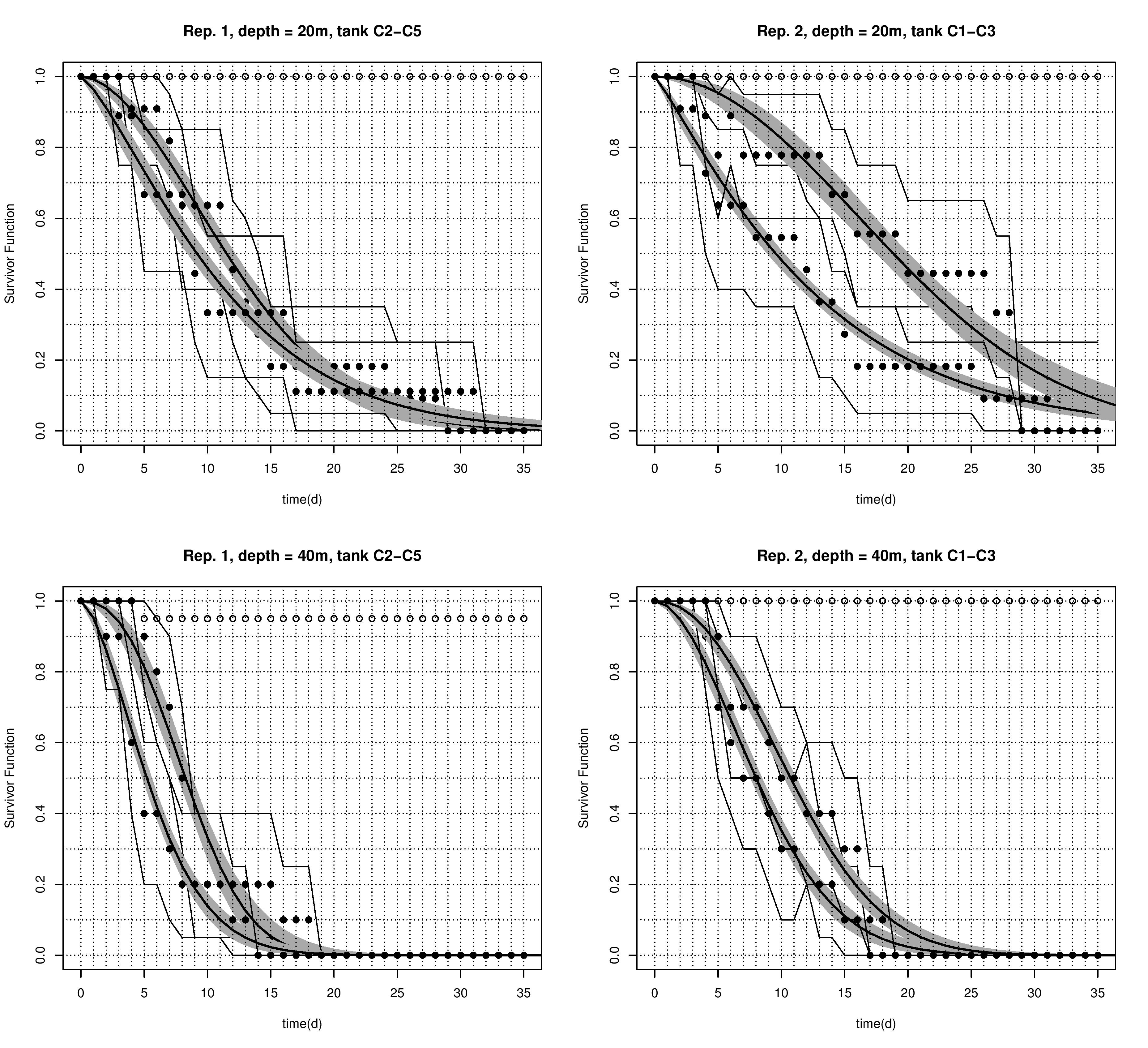


**Figure S6: Bayesian clustering analysis:**

A Bayesian clustering analysis has been done with the nine microsatellite loci with the STRUCTURE software [1]. We used an admixture model with correlation of allelic frequencies [2] and we tested K values from 1 to 5. Each run comprised a 200 000 iterations after a burn-in period of 100 000 and ten replicates for each run. The figure presents the membership probabilities for one replicate for K = 2 to 5. Samples are grouped according to the origin of the samples (1: experimental 20 m, 2: experimental 40 m; 3: 40 m *in situ*; 4: 20 m *in situ*). No structure was evidenced whatever the K value: all individuals displayed membership probabilities equally shared between clusters.

1. Pritchard JK, Stephens M, Donnelly P. 2000 Inference of Population Structure Using Multilocus Genotype Data. *Genetics* **155**, 945 –959.

2. Falush D, Stephens M, Pritchard JK. 2003 Inference of population structure using multilocus genotype data: linked loci and correlated allele frequencies. *Genetics* **164**, 1567–87.


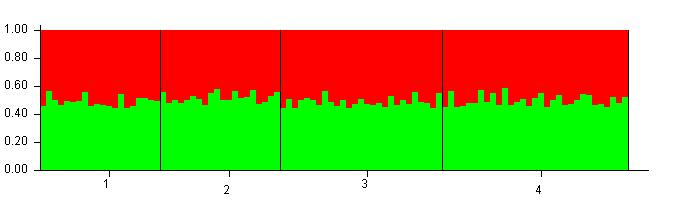


**K=2**

**K=3**


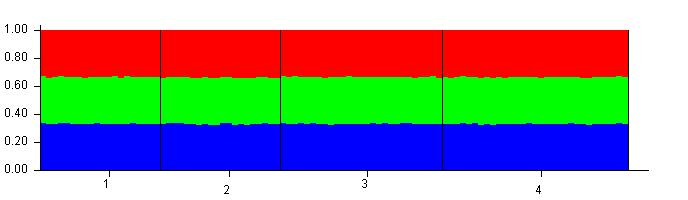


**K=4**


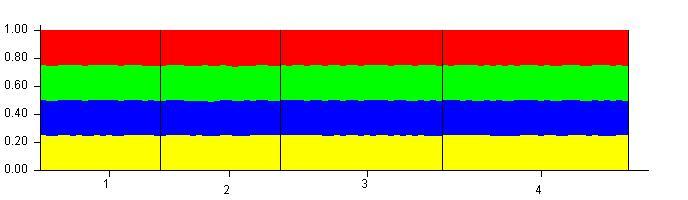


**K=5**


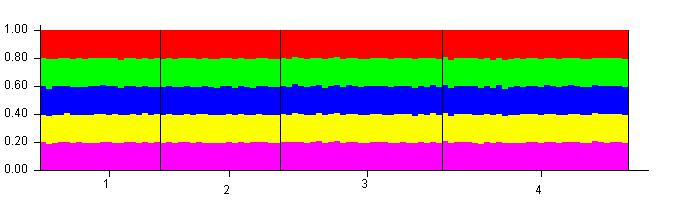

Supplement: Microsatellite genotyping : locus characteristics and PCR conditions Table S1: amplification conditions and origin of the nine microsatellite loci Table S2: microsatellite diversity by depth Table S3 : results of the Analysis of Molecular Variance with nine microsatellite loci. Figure S1: protocol o [file rsos140493supp1.doc]
